# Sharing the motherload: A review and development of the CO–Parent conceptual model for early childhood obesity prevention

**DOI:** 10.1111/obr.13853

**Published:** 2024-10-17

**Authors:** Konsita Kuswara, Vanessa A. Shrewsbury, Jacqui A. Macdonald, Alexandra Chung, Briony Hill

**Affiliations:** ^1^ The Centre of Research Excellence in Translating Early Prevention of Obesity in Childhood (CRE EPOCH‐Translate), Charles Perkins Centre University of Sydney Camperdown New South Wales Australia; ^2^ School of Health Sciences, College of Health, Medicine and Wellbeing The University of Newcastle Callaghan New South Wales Australia; ^3^ Food and Nutrition Research Program Hunter Medical Research Institute New Lambton Heights New South Wales Australia; ^4^ Centre for Social and Early Emotional Development, School of Psychology Deakin University Geelong Victoria Australia; ^5^ Centre for Adolescent Health Murdoch Children's Research Institute Parkville Victoria Australia; ^6^ Department of Paediatrics University of Melbourne Parkville Victoria Australia; ^7^ School of Public Health and Preventive Medicine Monash University Melbourne Victoria Australia

**Keywords:** conceptual model, fathers, mothers, pediatric obesity

## Abstract

Fathers remain under‐represented in early childhood obesity prevention research and interventions, despite growing evidence that paternal biopsychosocial factors and behaviors from pre‐ and post‐conception can influence lifelong offspring health. Informed by a literature review of high‐quality evidence, “CO–Parent” (childhood obesity–Parent) is a new conceptual model underpinned by couple interdependence theory and a socioecological framework. Literature was searched for the concepts parental AND weight‐related behaviors AND child weight or weight‐related behaviors, in databases including MEDLINE, PsycINFO, Global Health, Scopus, and SocINDEX. Prior evidence syntheses were prioritized as source data to inform model development. “CO–Parent” illustrates the interdependent and independent effects of maternal and paternal weight, weight‐related behaviors, and well‐being, across preconception, pregnancy, postpartum, and the early years on child weight‐related behaviors and weight up to age five. The influences of public policy, social, environmental, economic, community, and other complex modifiable mediating factors are included in the model. The “CO–Parent” conceptual model paves the way for a paradigm shift by recognizing fathers as key figures in early childhood obesity prevention initiatives, encouraging them to “share the motherload.” It highlights both the independent and interdependent roles fathers play in the epidemiology of obesity starting from preconception. CO–Parent also provides the foundations necessary to guide future theory and research to be more inclusive of fathers to further understanding of the independent and interdependent influences of parents in early childhood obesity prevention.

**Abbreviations**
CFIs: Couple‐focused interventions
COM‐B: Capability, opportunity, and motivation behavior framework
CO‐Parent: Child obesity‐Parent model
EDEN: EndObesity consortium
EPOCH: Exploring perinatal outcomes among children

## 1 INTRODUCTION

The first 2000 days of life, spanning conception to age 5, is a critical window for early obesity prevention as both biology and behavior are most amenable to change.^1^ Public health initiatives to prevent obesity in early life have largely focused on the mother as the primary contributor to growth and development and the role of fathers has received less attention.^2^ Yet, with growing evidence on paternal influence in the first 2000 days on their children's disease risks across the lifespan, there is a need to re‐evaluate early childhood obesity prevention approaches. Indeed, there is now evidence for both direct (e.g., epigenetic influences^3^) and indirect (concordance of health behaviors among couples^4^) paternal influences on child health.

Childhood health outcomes are nested within the broader socioecological contexts that shape child development.^5^ Thus, research, policy, and practice that take a continuous CO–Parent approach starting prior to conception have great potential to improve effectiveness and sustainability of early childhood obesity prevention initiatives.^6^ However, there are no contemporary, evidence‐based theoretical models to guide such approaches.

This review paper proposes a new conceptual model called the “Child Obesity–Parent (CO–Parent)” model. It outlines the independent and interdependent contributions of maternal and paternal weight; weight‐related behaviors; and wellbeing in preconception, pregnancy, postpartum, and the early years to children's weight and weight‐related behaviors in the first 2000 days of life. The intention is not to replicate work on factors influencing child health and weight outcomes, rather, we draw attention to the role of fathers. Specifically, we focus on the interdependence of father behaviors with maternal behaviors and how the roles of both parents are interwoven to influence child weight and weight‐related behaviors. A theoretical model of causality is vital if preventative measures are to be systematically tested and evaluated, and if understanding of independent and interdependent parental contributions to pediatric obesity is to be iteratively refined.^7^

## 2 METHOD

### 2.1 Definitions

**FIGURE 1:**
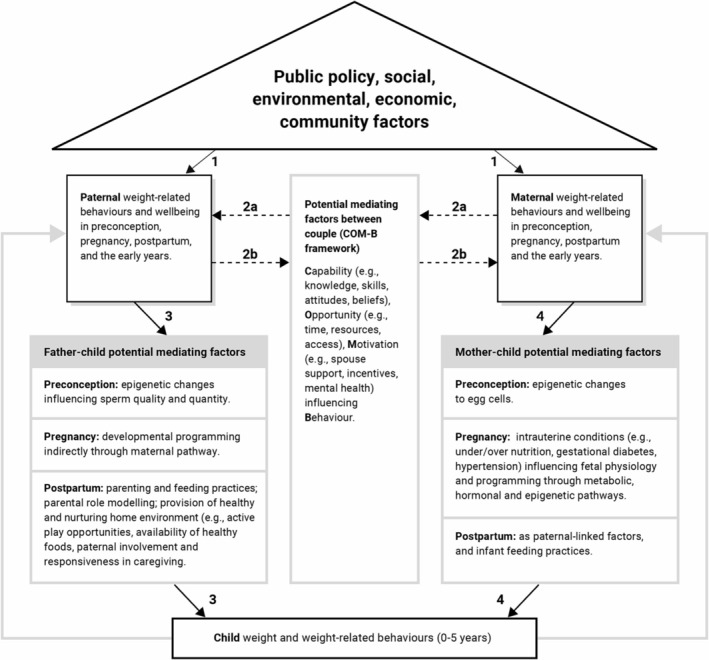
CO–Parent conceptual model for early childhood obesity prevention. *Key: Black boxes and triangle: components of the model including paternal, maternal, and child factors, along with ecological influences on health. Gray boxes: potential mediating factors between: father–child (left), mother–child (right), couple (center), in preconception, pregnancy, postpartum, and early childhood. Arrows: 1 – overarching influence of social, economic, environmental, and community factors on parental weight‐related behaviors and well‐being. 2a and 2b – interdependent (partner) effects of weight‐related behaviors and well‐being between couple across preconception, pregnancy, postpartum, and the early years. Dashed line represents the effect of paternal/maternal behaviors on their partner's outcomes (but not their own outcomes) through potential mediating factors. 3 and 4 – independent effects of paternal* (3) *and maternal* (4) *weight‐related behaviors and well‐being on child's weight and weight‐related behaviors across preconception, pregnancy, postpartum, and the early years. Overall, the child's weight and weight‐related behaviors (0–5 years) are influenced by both independent and interdependent effects of the biological mother and father, and parental figures. Gray arrow – potential influence of child's weight‐related behaviors on parental weight‐related behaviors and well‐being.*

The CO–Parent conceptual model (Figure 1) encompasses the preconception, pregnancy, and postpartum periods and the subsequent early years as distinct critical and interrelated windows for child health and development. The preconception period (incorporating interconception, that is, the period between pregnancies) is commonly considered to be the time when one is planning a pregnancy, and can also incorporate unplanned pregnancies (up to 50% of all pregnancies) by taking a population approach.^8^ Pregnancy includes the time from conception to delivery. We define postpartum and the early years as the time from delivery to 5 years post‐birth, when a child's weight‐related behavior patterns are developing.^6^ Weight‐related behaviors include diet, physical activity, sedentary behavior, and sleep. We use the term “weight‐related behaviors” for ease of narrative and do not wish to perpetuate the narrative of blame for individual responsibility of behaviors. These are considered core behavioral mechanisms and outcomes for obesity prevention interventions in early childhood.^9^ For the purposes of this paper, well‐being encompasses both body weight and psychosocial factors linked to physical health (including weight) such as depression, anxiety and stress symptoms, and body image.^10^

### 2.2 Method for model development

Conceptual model development was informed by Brady et al.’s three steps: (1) selecting resources for idea generation; (2) identifying potential factors for inclusion; and (3) selecting relevant factors for the model.^11^ Step 1 involved a literature search. Based on the relationships proposed in a draft of the CO–Parent model, we developed a PICOS/PECOS formulated literature search strategy with synonyms for the following terms: parental (maternal, paternal, and couple) AND weight‐related behaviors (diet, physical activity, sedentary behavior, and sleep) AND child weight or weight‐related behaviors, non‐restricted by publication date. Paternal and maternal relationships encompassed both biological parents and “parental figures,” although most studies did not differentiate between the two. The databases searched included MEDLINE, PsycINFO, Global Health, Scopus, and SocINDEX up to May 2022, updated in August 2023. The search results were screened and, in the process, relevant studies were mapped against the various hypothesized pathways in the model in a tabulated format to guide the first iteration of the model. Evidence syntheses (e.g., umbrella reviews, systematic reviews and meta‐analyses, and narrative reviews), published in English were prioritized as source data and were used to determine the face validity of the CO–Parent model proposed in Figure 1. Where evidence syntheses were lacking, primary studies were used. Steps 2 and 3 were conducted simultaneously, with model refinement and additional screening of literature occurring iteratively. To identify potential factors for inclusion and determine which should be incorporated in the model, we drew upon the interdependence model of couple influence^10^ and the Social Ecological Model of Health (described in the succeeding text).^5^ The literature review identified complexities, captured in the gray boxes of the model (Figure 1), via an iterative deliberative process of model refinement to reflect potential mediators of the proposed relationships. Additional targeted literature searches were conducted to explore the evidence on potential mediators. Our intent was not to capture a systematic and complete review of the literature but to demonstrate the rationale for model development with current evidence. We also drew upon extant literature as evidence to support links in the model that are novel.^12^

### 2.3 Model description and conceptual underpinning

The CO–Parent conceptual model (Figure 1) illustrates potential interdependent (arrows 2a and 2b) and independent (paternal arrows 3, maternal arrows 4) influences of maternal and paternal weight‐related behaviors and well‐being on each other, and on their offspring's weight and weight‐related behaviors. Parental influences include biological parents and parental figures. The potential influence of child's weight‐related behaviors on parental weight‐related behaviors and well‐being is acknowledged (gray arrow) but is out of scope to discuss in this paper. This model spans across preconception, pregnancy, postpartum, and the early years up to 5 years of age, with corresponding potential modifiable mediating factors (gray boxes). The model acknowledges the overarching influences of the Social Ecological Model of Health (triangle, arrows 1).^5^

**FIGURE 2:**
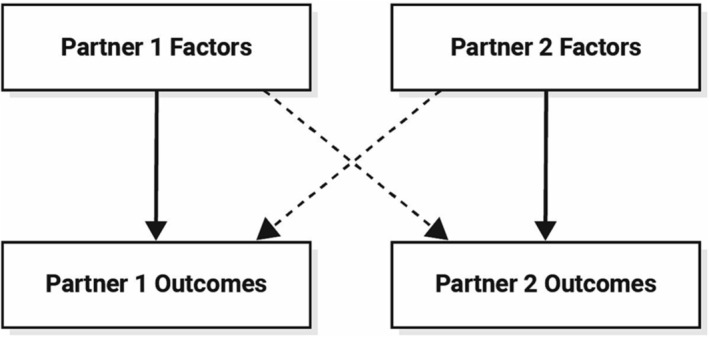
Example of a simple couple interdependence model (adapted from Lewis, McBride, Pollak, Puleo, Butterfield, and Emmons^10^). Key: *factors – any modifiable or non‐modifiable characteristic with potential to affect specified outcomes. Solid arrows – represents the possibility that individual members of the couple influence their own outcomes (e.g., thoughts, behaviors, and health wellbeing) with little influence from their partner. Dashed line – represents the possibility that each individual member of the couple influences their partner's outcomes but not their own outcomes. Also called partner effects. Combining dashed and solid arrows – represents the possibility of joint influence where an individual's outcomes are determined by both their own and their partners factors. Also called joint effects or mutual joint effects.*

CO–Parent is underpinned by principles of interdependence theory,^10^ which considers dyadic processes in understanding couple behavior and its implication for health promotion/disease prevention.^13^, ^14^ Interdependence is essential for a comprehensive understanding of human behavior and refers to the process by which interacting individuals influence each other's outcomes (e.g. behaviors, health, and well‐being).^15^ Figure 2 provides an example of a simple couple interdependence model, which informed the structure of the CO–Parent model.^10^ It recognizes the complex interactions within the couple unit enabling each individual to influence their own health and behaviors (called independent effects in Figure 1), and also the influences of their partner (called interdependent effects in Figure 1).

The CO–Parent model also incorporates the overarching context of the Ecological Model of Health (Figure 1, triangle),^5^ highlighting how individual health and health behaviors are shaped by interpersonal, social, community, economic, and public policy factors. By doing so, we acknowledge the social determinants of health, recognizing that the conditions in which people are born, grow, work, live, and age, as well as the systems that shape these daily life conditions profoundly influence their health and well‐being.^16^ Framing the model within a socioecological approach is essential to mitigating the blame and stigma placed upon individuals for imposing that they are entirely responsible for their health behaviors and their pregnancy, birth, and offspring outcomes, without acknowledging broader factors that influence health.^17^

## 3 RESULTS

In this section, we present evidence on each component of the CO–Parent model (Figure 1).

### 3.1 Wider influences of public policy, social, environmental, economic, and community factors

Consistent with the Social Ecological Model of Health,^5^ the CO–Parent model recognizes that individual health behaviors are enacted within the constraints of socioecological influences, including public policy, social, environmental, economic, and community factors, represented by the arrows labeled “1” (Figure 1). These factors shape the conditions in which people carry out their daily lives. Access to quality education; the opportunity to earn an income; and adequate living conditions including safe neighborhoods, affordable housing, and healthy food environments are all fundamental to health and well‐being, and all have an impact on weight and weight‐related behaviors.^16^ Yet, these circumstances are experienced differently by different populations, leading to socioeconomic inequities in the distribution of health and illness.^16^ Adults who experience greater socioeconomic disadvantage are at increased risk of unhealthy weight‐related behaviors and overweight and obesity, compared to adults who experience relatively less disadvantage.^16^, ^18^ The Foresight Report on obesity described the complex drivers of obesity across the population spanning these broader influences.^19^ Many of these drivers apply to the reproductive life stage.^17^ For example, a study of Dutch women affected by overweight or obesity across preconception, pregnancy, and 1‐year postpartum indicates that everyday diet and movement behaviors are influenced by features of the built environment (e.g., bike lanes, parks, and pedestrian paths), public policy supporting access to healthy food or physical activity, and workplace policies that support work–life balance.^20^ Young Australian men have reported similar barriers to adopting healthy behaviors including difficulties accessing healthy foods and work commitments that reduce time availability for healthy food preparation and exercise.^21^ Social influences such as family structure and intergenerational pathways (e.g., grandparents) also have significant impacts on child health.^22^ A growing body of research demonstrates that family structure has an influence on the resources available to families and can in turn affect child health outcomes, including overweight and obesity and mental health.^23^, ^24^, ^25^ It is therefore important that individual and family health behaviors are considered within broader socioecological contexts.

### 3.2 Interdependent parental influences on each other's weight‐related behaviors and well‐being

The CO–Parent model highlights potential interdependent influences on parents' weight‐related behaviors and well‐being. That is, fathers' influences on the mother and vice‐versa occurring from preconception through to postpartum and the early years (Figure 1, arrows 2a, 2b).

Evidence from primary studies (in the absence of reviews) exploring the interdependent relationships between couple weight‐related behaviors across the reproductive phase is emerging and centers mainly on pregnancy and postpartum. For example, in a qualitative study involving couple dyads, there was high concordance for reported dietary behaviors during pregnancy, but less so for physical activity behaviors.^4^ Couple‐dyad behaviors also appear to be associated with maternal outcomes in pregnancy. For instance, a longitudinal couple‐dyad study of mother–father dietary behaviors and associations with gestational weight gain showed that concordance (unhealthy dietary behaviors in both parties), or discordance (healthy behaviors in one parent but not the other), led to greater weight gains than that experienced by women who consumed a healthy diet together with their partner.^26^ Findings from a pilot randomized trial of a physical activity intervention in postpartum couple dyads showed a positive effect on maternal physical activity level when they collaboratively planned physical activity together with their partner.^27^ These findings are consistent with studies on other behaviors, such as smoking, in the reproductive phase,^28^ which further substantiates the interdependent relationship proposed in our model.

In adults living with overweight or obesity (not specifically preconception, pregnant, or postpartum), evidence for dyadic influences on weight outcomes supports findings in pregnancy and postpartum/early years. For example, in a weight loss randomized intervention study, inclusion of partners in the intervention facilitated weight loss, while the reverse occurred when partners were not involved.^29^

As illustrated in Figure 1 (center gray box), interdependence between couple's weight‐related behaviors and well‐being is potentially mediated by psychosocial factors identified within the Capability, Opportunity, and Motivation‐Behavior (COM‐B) framework.^30^ For example, a study in adolescent and young adult new parents reported that interdependence behaviors for diet (e.g., unhealthy eating behaviors) may be stronger when one partner reports more power in the relationship (e.g., control over food purchasing).^29^ Relationship power could reflect a form of motivation where one partner changes their behavior to maintain a harmonious relationship.^10^ Alternatively, when one parent exerts greater control in ensuring a healthy home food environment, this may provide greater opportunity for their partner to eat healthy foods.^29^ Over time, this may translate into greater capability (attitudes and skills) for both parents to eat a healthier diet. The same study found no partner influence effects for exercise, potentially because exercise is more difficult to do with your partner in the early days of parenthood,^29^ suggesting the importance of opportunity as a mediator in influencing weight‐related behavior change.

A review of co‐parenting research highlights the interdependence between parents, demonstrating how their interactions as parents impact their well‐being and influence children's development.^31^ Supportive or concordant co‐parenting is associated with higher parenting self‐efficacy, which predicts higher parenting competence, and improved development outcomes in children, such as enhanced self‐regulation.^31^ Evidence from a randomized controlled trial further indicates that co‐parenting interventions (e.g., conflict management, communication, and mutual support) during pregnancy and postpartum lead to healthier maternal postpartum weight.^32^ Additionally, recent cross‐sectional studies suggest that supportive co‐parenting is linked to healthier child weight‐related behavior including better sleep^33^ and reduced picky eating.^34^

Overall, there is emerging evidence from primary research studies in pregnancy and postpartum to support the interdependent influences illustrated in the CO–Parent model (Figure 1). Hence, we hypothesize that dyadic interdependence is an important factor to consider in predicting, and potentially improving, parental weight‐related behaviors across the preconception, pregnancy, postpartum, and the early years periods, albeit further research is needed. However, despite recommendations released almost three decades ago for a greater focus on reproductive health interventions targeting couples as a unit, this has not become common in research or practice for interventions targeting weight‐related behaviors.^35^

### 3.3 Independent effects of parental weight‐related behaviors and well‐being on child weight‐related behaviors and weight, across preconception, pregnancy, postpartum, and the early years

The CO–Parent model proposes that during preconception, pregnancy, postpartum, and the early years, the weight‐related behaviors and well‐being (including obesity) of each parent influence their child's growth in‐utero, birth weight, and early childhood weight status (Figure 1, arrows 3 and 4). In the following sub‐sections, we present the evidence and potential mechanisms demonstrating independent effects on child weight‐related behaviors and weight by reproductive phase. Despite our attempt to synthesize the independent associations of each parent, many studies did not consider the interdependent influences (e.g., including key covariates in analyses), which should be considered when interpreting the findings. Further, there could be potential bi‐directional relationships between child and parents' weight‐related behaviors in the postpartum period and early years of life,^36^ (Figure 1, gray arrows), but it is beyond the scope of this paper to discuss these in detail.

#### 3.3.1 Preconception

Paternal and maternal health, behavior, genetic influences, and psychosocial factors in the preconception period affect their offspring's weight trajectory.

##### Paternal influences

In the preconception period, evidence of the independent effects of paternal health behavior and well‐being on children's weight or weight‐related behaviors was strongest for paternal obesity. A systematic review of observational studies suggests that paternal obesity in preconception (and pregnancy) is adversely correlated with several conception, pregnancy, neonatal, and early life health outcomes.^37^ Infants of fathers living with obesity had increased frequency of small‐for‐gestational‐age, or macrosomia (and therefore risk of higher weight in early childhood^38^), independent of maternal factors.^37^ Findings from the Danish Birth Cohort study further showed that both maternal and paternal obesity in the preconception period are independently linked to higher child weight at birth and at 7 years of age, although the maternal effect is stronger.^39^

Another recent systematic review of observational studies found limited and mixed evidence on the impact of paternal preconception diet and movement behaviors on their child's weight or weight‐related behaviors.^40^ Studies that examined a range of paternal preconception dietary exposures did not find associations with their child's birth weight but have found weak positive associations with other outcomes such as placenta weight, gestational age, head circumference, and lower newborn adiposity.^41^, ^42^ Not all studies adjusted for maternal factors.

In a study that investigated data from four European mother–offspring cohorts (which included data from fathers) participating in the EndObesity Consortium [EDEN, France; Elfe, France; Lifeways, Ireland; and Generation R, The Netherlands], various combinations of parental lifestyle patterns in preconception were associated with risk of child overweight after 5 years. In these analyses, the combination of factors in each cohort that contributed to explaining the most variance in risk of child obesity were characterized by paternal and maternal smoking, paternal and maternal obesity, low maternal diet quality, low paternal intake of vegetables, and low paternal diet quality. While sedentary behavior and physical activity were also assessed, these behaviors were not directly feature in these patterns.^43^

More evidence is available from animal studies, which reported that poor paternal preconception diet in mice (high fat, high sugar, low protein, and low micronutrients) was associated with increased risks of higher offspring adiposity, body weight, impaired glucose tolerance, and altered lipid metabolism.^44^ These studies further showed that paternal exercise training around conception offsets metabolic dysfunction in offspring.^44^ Although these findings cannot be extrapolated directly to humans, they strengthen the argument to consider paternal preconception health and health behaviors in the early prevention of childhood obesity.

##### Maternal influences

Preconception maternal obesity increases risk of numerous adverse outcomes, including difficulty conceiving, pregnancy and delivery complications, congenital anomalies, and breastfeeding challenges.^45^ Furthermore, maternal obesity is increasingly recognized as having a significant influence on many child factors, including body composition, cardiometabolic health, and potentially immune, infectious disease and neurocognitive outcomes.^46^ Data from the US National Longitudinal Survey of Youth show that children of mothers in higher weight trajectory groups (i.e., weight/weight change higher than recommendations across the child‐bearing period) compared with children of mothers in the lowest weight trajectory group had a twofold increase in obesity risk in childhood.^47^ However, this study did not adjust for paternal weight and numerous other important covariates.^47^

The influence of maternal preconception health behaviors on child obesity risk was reported in the previous section (see summary of data from the EDEN, Elfe, Lifeways, and Generation R studies earlier).^43^ Similar findings were found in a longitudinal analysis of the UK Southampton Women's Survey,^48^ which found that poorer diet quality trajectories from preconception and through pregnancy (assessed by maternal dietary intake) to age 8–9 years in the child (assessed by the child's dietary intake) were associated with higher child adiposity outcomes at age 8–9 years. In addition, preconception diet and nutrition can modify maternal and perinatal outcomes through other nutritional factors such as micronutrient deficiencies.^49^ Preconceptionally, maternal diet and physical activity are inextricably linked with psychosocial well‐being^10^; the potential impacts of maternal depression, anxiety, and stress before conception on offspring future health, including weight, cannot be ignored.

##### Parental genetic and epigenetic influences

The impact of parental preconception weight and weight‐related behavior on their child's growth in‐utero, weight at birth, and early childhood is influenced not only by genetic heritability^50^ but also by environmental conditions that induce epigenetic modifications.^51^ Reviews of human and animal studies indicate that high parental body mass index (BMI), unhealthy dietary patterns (high in sugar, fat, and processed foods), and stress in the preconception period are associated with epigenetic changes to the male and female gametes.^51^ These changes affect gene expressions resulting in dysregulation of obesity‐related metabolites including glucose, insulin, and lipids, and placental nutrient transport and inflammation, increasing the offspring's susceptibility to obesity.^51^, ^52^ For example, paternal mice fed a high‐fat diet produced offspring with early onset impaired glucose tolerance and insulin resistance, mediated through alterations in the sperm genome (non‐coding RNAs) and genetic expression profile.^53^ Epigenetic influences on obesity risks may be mitigated by healthy behaviors periconceptionally. For example, animal studies showed that paternal exercise in the preconception period could reprogram genetic transcriptions which improved placenta conditions and offspring metabolic regulations and body weight in infancy.^54^

In addition to influencing the parental genome, preconception weight‐related behavior of the father impacts the offspring indirectly through maternal uterine environments. Human and animal studies showed that seminal plasma has a role in shaping maternal pregnancy outcomes and offspring metabolic parameters. Epidemiological studies suggest that increased exposure to partner's semen could lower prevalence of preeclampsia and inflammation.^55^ In animal models, paternal seminal fluid deficiencies led to offspring with impaired glucose tolerance, hypertension, altered metabolic hormones, and obesity.^56^

Given that parental influence on offspring's health is evident before conception, the preconception period marks a key time for public health interventions promoting health behavior and weight. In particular, fathers should be included as part of a couple unit in preconception health promotion interventions to enhance child health outcomes.^57^

#### 3.3.2 Pregnancy

Prenatal growth, birth weight, early childhood weight status, and weight‐related behaviors are impacted by paternal and maternal influences during pregnancy to varying degrees.

##### Paternal influences

In addition to the legacy of paternal genetic and epigenetic contributions at conception to fetal development, there is evidence for paternal influence on the offspring's susceptibility to obesity. A recent longitudinal study found positive associations between paternal sedentary time, measured objectively in the first trimester, and their child's weight trajectory in the first year of life, in girls but not in boys.^58^ A potential mechanism is for this link to occur indirectly through maternal health behavior and well‐being (see previous section on interdependent influences). Fathers have a role in contributing practical, emotional, and economic support that can impact maternal health and well‐being. Consistent evidence from observational and intervention studies shows that supportive paternal involvement in pregnancy is linked to positive maternal perinatal behaviors such as improved antenatal care attendance, good nutrition, breastfeeding, reduced smoking and reduced alcohol consumption, and reduced odds of postpartum depression.^59^

Pregnancy is also an important period for the psychological preparation of parenthood, and fathers' involvement in pregnancy could support not only the mother but also their own transition to parenthood. A systematic review of qualitative studies with first time fathers showed that they experienced very little support in navigating psychological and practical challenges associated with fatherhood, impacting their abilities to engage and support their partner and child.^60^ However, greater involvement in pregnancy, for example through attending antenatal appointments or making shared decisions about pregnancy and baby care, is a strong predictor to a more engaged fatherhood over the long term.^61^ Despite the evidence of the benefits in involving fathers during pregnancy towards maternal and child health, targeted interventions to support fathers in the perinatal period remains a gap.^62^

##### Maternal influences

The fetus is vulnerable to the intrauterine conditions which can influence growth and future weight trajectory.^63^ Meta‐analyses show that fetal exposure to high maternal gestational weight gain (usually defined as exceeding the Institute of Medicine recommendations)^64^ or gestational diabetes increases the risk of infants born large‐for‐gestational age and with macrosomia, among other adverse maternal and infant outcomes, compared with women who gained weight within recommended levels or did not have gestational diabetes, respectively.^65^, ^66^ Furthermore, evidence from the US Exploring Perinatal Outcomes among Children (EPOCH) retrospective cohort study showed that fetal exposure to gestational diabetes was associated with a higher risk of above average BMI, and accelerated BMI growth trajectory, during childhood.^67^ Gestational weight gain is likely causally influenced by factors such as depressive symptoms, coping skills, and body image via impacts on diet and physical activity habits^68^; therefore, maternal psychosocial factors in pregnancy are also implicated in child obesity development.

It is well accepted that physical activity in pregnancy can have many positive effects on maternal health and well‐being, and at low intensity is not harmful to the fetus. A systematic review of observational studies examining the effect of physical activity on gestational weight change inconclusively indicated that physically active pregnant women gained less weight than inactive pregnant women.^69^ Similarly, a meta‐analysis of randomized controlled trials found that, in women with overweight or obesity, exercise reduced gestational weight gain and diabetes risk, but no evidence identified specific benefits or harms for infants.^70^

Evidence on the influence of maternal dietary‐related factors, sedentary behavior, and sleep in pregnancy, on offspring weight or weight‐related behaviors, is emerging. A 2021 individual participant data meta‐analysis found no evidence that antenatal diet or combined diet and physical activity interventions for women with a BMI of 25 kg/m^2^ or above reduced the risk of early child obesity.^71^ However, other evidence suggests that in pregnancy, maternal impacts on offspring obesity risk potentially occur via the development of early food preferences and possibly dietary patterns.^72^ A systematic review identified limited but consistent evidence that maternal ingestion of foods with specific flavors can expose the fetus to these flavors through the amniotic fluid, priming for acceptance of similarly flavored foods during re‐exposure in early childhood.^72^ There is also preliminary evidence that the maternal gut microbiome, which is influenced by many factors including diet, is linked with maternal obesity and excess gestational weight gain, gestational diabetes, pre‐eclampsia, and infant metabolic health and other infant outcomes.^73^

Another systematic review identified limited evidence showing a significantly higher proportion of babies born with macrosomia or larger abdominal circumference among mothers demonstrating sedentary behaviors.^74^ Additional systematic review evidence, investigating the associations between maternal sleep behaviors in pregnancy (i.e., night‐time sleep duration, sleep quality, night awakenings, and daytime nap duration) and gestational eating behaviors, physical activity, and weight gain identified a significant association only between good sleep behaviors and higher physical activity levels in pregnancy.^75^ Animal research evidence on the effects of maternal sleep deprivation suggests that sleep disturbances in pregnancy could adversely affect mother and offspring by affecting behavioral, hormonal, electrophysiological, metabolic, and epigenetic factors.^76^

#### 3.3.3 Postpartum and the early years

In the postpartum period, the transference of weight‐related behaviors between family members and the influence of psychosocial factors are the key mechanisms of interest.

##### Paternal influence

In the early postpartum period, meta‐analytic evidence shows that paternal involvement in breastfeeding interventions resulted in an increase in exclusive breastfeeding at 6 months of age.^77^ Increased paternal support in breastfeeding is hypothesized to have a positive effect on maternal motivation, self‐efficacy, and capacity to maintain breastfeeding.^77^ This is important as breastfed infants have reduced odds of overweight/obesity in childhood and adulthood.^78^ After the infancy period, longitudinal evidence has shown positive correlation between fathers and their child's dietary patterns throughout the first 5 years of age.^79^ There is evidence primarily from observational studies,^80^ showing a modest positive relationship between fathers and their child's physical activity levels, including higher intensity physical activity. Limited evidence from cross‐sectional studies has found mixed results on paternal influence on young children's sedentary behavior.^81^

In regards to child sleep, systematic review evidence suggests that the quality of father–child interactions is linked with child's sleep outcomes.^82^ For example, poorer paternal mental health was associated with higher child sleep problems, although the evidence is cross‐sectional and derived from self‐report measures.^82^ In contrast, higher paternal involvement in daytime and bedtime caring, as well as emotional support to child, seems to have a positive impact on child's sleep quality.^82^

Paternal parenting practices and role modeling around food and physical activity likely explain, in part, their influence on child's diet and physical activity behaviors, and adiposity risk. A narrative review of mostly cross‐sectional studies demonstrated consistently that child dietary intake and eating behavior in the first 5 years of life were influenced through paternal role modeling and feeding practices.^83^ The review suggests that fathers were more likely than mothers to use less responsive feeding practices such as restricting or pressuring child to eat, and using food to regulate emotions, which was associated with reduced child ability to self‐regulate their eating behaviors.^83^ There is also emerging evidence to indicate that paternal depression may influence children's eating behaviors such as responsiveness to food and emotional overeating.^84^ In primary school age children, there is evidence that paternal role modeling of physical activity and exercising together with the child significantly mediated the intervention effect on children's physical activity level.^85^

##### Maternal influence

Continuing on from pregnancy research demonstrating that flavor preferences develop from the fetal stage,^78^ there is moderate level systematic review evidence that some flavors ingested by breastfeeding mothers have a time‐dependent effect on flavoring breastmilk, also potentially influencing infant taste preferences and dietary intake.^72^ Maternal body dissatisfaction may also influence breastfeeding establishment and maintenance^86^; sustained breastfeeding is associated with lower child obesity risk.^87^

At the complementary feeding stage, parental dietary intake^88^ and role modeling of eating and feeding practices^89^ can influence their child's dietary intake, food preferences, and obesity risk. Systematic review and meta‐analytic evidence show that children receiving some types of responsive complementary feeding have lower BMIs in early childhood compared with children exposed to non‐responsive or traditional complementary feeding practices.^90^ There is also an emerging body of literature explicating the impact of maternal postpartum psychosocial well‐being (including depression and anxiety) on child obesity risk.^91^ Not only is psychosocial and physical health intertwined, but further impacts on child weight are proposed to occur via influences on attachment security and establishment of parent–child feeding interactions. In terms of direct impacts on diet, evidence from a systematic scoping review of pre‐schoolers' dietary intakes confirmed that parental (data collected mainly from mothers) dietary intakes were positively associated with child dietary intakes in all studies.^88^

Mothers also play a role in transferring physical activity behaviors to their children and family.^92^ Systematic review evidence supports a weak positive relationship between parent and child physical activity regardless of child age, the gender of the parent–child dyad, and type of physical activity.^93^ Indeed, consistent evidence from a systematic review of observational studies indicates positive associations between maternal role modeling and young children's physical activity and sedentary behavior.^94^

In the postpartum period, sleep‐related behaviors of infants and parents appear to influence each other in a bidirectional and dynamic relationship.^95^ Short sleep duration is associated with obesity risk in early childhood, but less is known about the influence of other dimensions of sleep including quality, efficiency, and bed/wake times.^96^ Short sleep duration is also associated with obesity risk in adults but less strongly than it is for children.^97^ Potential mechanisms for this association include energy homeostasis, insulin resistance, and beta‐cell function.^97^

## 4 DISCUSSION

The CO–Parent conceptual model advances our knowledge and application of theory for the prevention of child obesity by acknowledging both maternal and paternal influences—and their interdependence—on child health prior to and during the first 2000 days of life. This shifts the current paradigm where the focus is on mothers as primarily responsible for child health to emphasizing the fundamental, not optional, role of fathers in early childhood obesity prevention. This is important because very few preconception and pregnancy interventions to date, that are designed to prevent overweight and obesity in children, involve participants' partner or social network.^98^ CO–Parent provides the conceptual foundations necessary for future thinking, research, policy, and practice to create more effective and sustainable early childhood obesity prevention interventions.

Taking a dyadic approach in promoting health in preconception, pregnancy, postpartum, and early childhood acknowledges the broader contexts that influence women's health and parenting practices, with onflow effects for childhood weight‐related behaviors and well‐being, as our model depicts. This approach also lessens the social motherload—the blame and burden society places upon women for children's health outcomes.^99^ Others have called for a similar approach, finding feasibility and acceptability of involving both parents in interventions to support healthy weight‐related behaviors during preconception and pregnancy. Benefits to delivering health interventions to couples or dyads include better outcomes and increased engagement, and positive “ripple effects,” whereby healthy behavioral changes achieved by one individual extend to the other.^100^ Challenges include difficult relationship dynamics, asynchronous schedules, differences between dyad members' motivation, engagement and success, and negative “ripple effects,” whereby the difficulties experienced by one individual extend to the other dyad member.^100^ Furthermore, Couple‐Focused Interventions (CFIs) in the reproductive health area more broadly, while still relatively rare, have been found to be equally or more effective in achieving immediate and longer‐term health outcomes, when compared to interventions targeting individuals.^101^ Evidence on the influence of fathers on child obesity risk is sparse compared to the evidence around mothers. There is also limited epidemiological evidence on the interdependence of mother–father behaviors in shaping child weight‐related outcomes. For example, the potential dose effects of consistent influence from two parents, the heterogenous effects from inconsistent influences between parents, and the effects of differential changes in individual parent's behaviors over time are all further considerations. Hence, there is a need for research, policy, and practice to better address a father's role, both directly and indirectly, in influencing the risk of child obesity as shown in Box 1.

Box 1 **Future research, policy, and practice that can be guided by CO–Parent.**

Research

- Collect relevant data from fathers/partners in addition to data from mothers.
- Routinely adjust for the “other parent” in analyses to fully elucidate independent effects.
- Consider potential maternal biases when developing research questions
- Guide future systematic reviews

Policy

- Invest in targeted health promotion and prevention initiatives for boys and men in routine primary health care, alongside initiatives for girls and women
- Greater investment in flexible paid parental leave schemes which can be shared with fathers and promote uptake of the schemes particularly in male‐dominated workplaces
- Encourage flexible work arrangements for both parents

Practice

- Enhance conversations that consider a woman's support system during routine care in preconception, pregnancy, and postpartum
- Improve access to parenting preparation programs for both parents
- More programs targeting couples as a unit in preconception through to the early years, where appropriate,^4^ with evaluation of the independent and interdependent parental effects on child health outcomes.
- Naming and scope of clinical practice guidelines and services to be more inclusive of fathers/partners

There is significant opportunity to invest in obesity prevention during the preconception period with a focus on the role of fathers as well as mothers.^3^ Australia's National Obesity Strategy implicitly recognizes the role of both parents by citing the need to support prospective parents before pregnancy occurs and provide guidance to new parents to maintain healthy weight‐related behaviors.^102^ Preconception health (for both those planning pregnancy and for all people when taking a life course approach) is especially important given around 40% of pregnancies worldwide are unplanned.^103^ Building on this, future policy reform could include health interventions for adolescent and adult males in routine primary care and explicit support for fathers built upon greater investment in men's preconception health^57^ and paid parental leave schemes. Evaluation of Australia's Paid Parental Leave scheme complementary Dad and Partner Pay initiative found associations with improved maternal mental health, the flow on effects of which included improved maternal–infant interactions and better child health outcomes.^104^ International evidence also indicates that paid parental leave is associated with better infant and child health outcomes.^105^

While the CO–Parent model provides a solid foundation on which to guide current research, it is open to future development and improvement. For example, the diversity of family structures in contemporary society should also be considered. Adaptations to CO–Parent will likely be required as evidence continues to evolve on the roles of other parents and caregivers in different family contexts including single‐parent, same‐sex parent families, and extended family groups. This includes providing practical, emotional, knowledge, or financial support at various stages of the first 2000 days. Future iterations of CO–Parent should also consider more broadly the impacts of mental health and well‐being on behaviors, weight, and factor interdependence across mothers, fathers, and children. Further research in each of these areas is required to better understand the context‐specific mechanisms that drive child obesity outcomes and develop the necessary and appropriate policy and practice interventions. There is also a need to better understand the mechanisms linking parental weight‐related behaviors in preconception and pregnancy with child weight, and the role of parental weight or weight gain.^72,107^ This is important to identify the most effective window for intervention and to shift the focus from weight alone to modifiable health behaviors as well as broader socioecological factors that can be targeted. Finally, future iterations should continue to advance from conceptual understanding and provide explicit pathways that form testable hypotheses and evaluate the strength or level of evidence supporting the model or its component pathways. This will help to systematically build the evidence base in a way that informs targeted intervention development.

## 5 CONCLUSION

Using the CO–Parent model to guide future research, policy, and practice has the potential to reduce risk of obesity in early childhood. There is a need for greater sharing of the “motherload” for obesity prevention to highlight and leverage the fundamental role of fathers. Future epidemiological research needs to include paternal factors so that a more accurate perspective of the importance of maternal and paternal factors, and their interdependence, can be understood. Interventional research to prevent obesity also needs to have a greater focus on the role of both parents recognizing that contemporary parenting is increasingly a shared responsibility.

## AUTHOR CONTRIBUTIONS

All authors contributed to conceptualizing the study. Konsita Kuswara, Vanessa A. Shrewsbury, and Briony Hill conducted the literature searches. Konsita Kuswara, Vanessa A. Shrewsbury, Briony Hill, and Alexandra Chung drafted the figures and sections of the manuscript. All authors (Konsita Kuswara, Vanessa A. Shrewsbury, Briony Hill, Alexandra Chung, and Jacqui A. Macdonald) provided intellectual content, edited the manuscript, approved the final version for submission, and agree to be accountable for all aspects of the work.

## CONFLICT OF INTEREST STATEMENT

JM reports roles on the Scientific Advisory Group for the Australian Longitudinal Study on Male Health and as a Convenor of the Australian Fatherhood Research Consortium.
